# Microbial diversity in four Mediterranean irciniid sponges

**DOI:** 10.3897/BDJ.12.e114809

**Published:** 2024-01-19

**Authors:** Katerina Skaraki, Christina Pavloudi, Thanos Dailianis, Jacques Lagnel, Adriani Pantazidou, Antonios Magoulas, Georgios Kotoulas

**Affiliations:** 1 Institute of Marine Biology, Biotechnology & Aquaculture, Hellenic Centre for Marine Research, Heraklion, Crete, Greece Institute of Marine Biology, Biotechnology & Aquaculture, Hellenic Centre for Marine Research Heraklion, Crete Greece; 2 Department of Ecology & Systematics, Faculty of Biology, National & Kapodistrian University of Athens, Athens, Greece Department of Ecology & Systematics, Faculty of Biology, National & Kapodistrian University of Athens Athens Greece; 3 European Marine Biological Resource Centre (EMBRC-ERIC), Paris, France European Marine Biological Resource Centre (EMBRC-ERIC) Paris France; 4 INRAE, UR1052, Génétique et Amélioration des Fruits et Légumes (GAFL), Centre de Recherche PACA, Montfavet, France INRAE, UR1052, Génétique et Amélioration des Fruits et Légumes (GAFL), Centre de Recherche PACA Montfavet France

**Keywords:** sponge metagenome, marine metagenome, amplicon sequencing, 454 GS FLX Titanium, pyrosequencing, eastern Mediterranean

## Abstract

This paper describes a dataset of microbial communities from four different sponge species: *Irciniaoros* (Schmidt, 1864), *Irciniavariabilis* (Schmidt, 1862), *Sarcotragusspinosulus* Schmidt, 1862 and *Sarcotragusfasciculatus* (Pallas, 1766). The examined sponges all belong to Demospongiae (Class); Keratosa (Subclass); Dictyoceratida (Order); Irciniidae (Family). Samples were collected by scuba diving at depths between 6-14 m from two sampling sites of rocky formations at the northern coast of Crete (Cretan Sea, eastern Mediterranean) and were subjected to metabarcoding for the V5-V6 region of the 16S rRNA gene.

## Introduction

Porifera (sponges) is one of the oldest metazoan Phyla (Müller 2003), comprising species very important for the shaping and functioning of benthic ecosystems. Sponges maintain symbiotic microorganisms that, in many cases, can comprise up to 40% of their biomass (Vacelet and Donadey 1977, Hentschel et al. 2003). Microbes associated with sponges belong to phylogenetically diverse phyla of Prokaryotes and Eukaryotes (Taylor et al. 2007). The main objective of this paper was to study the microbial diversity associated with a group of Porifera species that exhibit certain characteristics of interest, such as inhabiting an oligotrophic environment, i.e. the South Aegean Sea (east Mediterranean) and being difficult to identify taxonomically while, at the same time, considered as model taxa in host-associated microbiological studies (Hardoim and Costa 2014, Pita et al. 2013), like species of the family Irciniidae. In addition, by collecting samples from various species and tissues, we were able to identify if there is host and/or tissue specificity in the sponge microbiome.

### Value of the dataset

This dataset includes microbial taxa inhabiting the demosponges *Irciniaoros*, *I.variabilis*, *Sarcotragusspinosulus* and *S.fasciculatus* and contributes to the ongoing efforts of the Ocean Biogeographic Information System (OBIS) which aims at filling the gaps in our current knowledge of the world's oceans. The dataset has been also published in GBIF (Skaraki et al. 2023).

## Methods

### Sampling

Sponge samples were collected in 2009-2010 by scuba diving from two sampling sites of rocky formations at the northern coast of Crete (Cretan Sea, eastern Mediterranean). Specimens of *Sarcotragusspinosulus*, *Irciniaoros* and *I.variabilis* were collected from Alkes along with a seawater sample (2 litres), whereas samples of *Sarcotragusfasciculatus* were collected from Elounda. Samples were transferred to the laboratory in a cooler with ice packs. Upon arrival, sponge samples were preserved in 97% ethanol and stored at -20°C prior to DNA extraction; the seawater sample was filtered through a Whatman 0.45 um mixed cellulose ester membranes filter, which was stored at -80°C prior to DNA extraction. Sponge samples were identified both morphologically, as well as by molecular markers (18S rRNA and COI).

#### Geographic range

The dataset's geographical range includes two locations in Crete (Greece) (Table 1).

### Sample processing

For each sponge specimen, mesohyl and ectosomal tissue samples (10-15 mg each) were cut and proceeded separately. DNA from those tissues was extracted using DNeasy Blood & Tissue™ kit (Qiagen) following the manufacturer’s guidelines. DNA from the filter was extracted according to a freeze–thaw–boiling protocol of Riemann and Middelboe (2002). DNA extracts were stored at -20°C prior to amplification.

#### Amplification & Sequencing of 16S rRNA

The V5-V6 hypervariable region (~ 280 bp) of the 16S ribosomal RNA (rRNA) gene was amplified using the degenerate universal primers 802F: 5'-GGATTAGATACCCBNGTA-3' (originally designed as reverse primer by Claesson et al. (2009)) and 1027R: 5'-CGACRRCCATGCANCACCT-3' (Claesson et al. 2009) that target Bacteria and Archaea. In order to pool samples prior to sequencing, the primers contained a 10 bp length of multiplex identifiers (MIDs) between the 454 adaptor A (forward primer) and B (reverse primer), according to Roche. PCR reactions were performed in a total volume of 25 ul containing 0.3 uM of each primer, 0.3 mM dNTPs, 0.5 U KAPA high-fidelity HotStart DNA polymerase (Kapa Biosystems), 2.0 mM MgCl_2_ and approximately 20 ng of template DNA. PCR reactions were carried out in a thermal cycler (Bio-Rad, MyCycler) under the following conditions: initial denaturation at 95°C for 5 min; followed by 25 cycles of denaturation at 98°C for 20 s, annealing at 55°C for 15 s and extension at 72°C for 60 s, followed by a final extension step at 72°C for 5 min. PCR amplicons were checked by electrophoresis in 1% agarose gel. Amplicons were purified by Agencourt AMPure XP purification system (Beckman Coulter Genomics) and quantified with Quantit PicoGreen dsDNA (Invitrogen) in a Quantiflour fluorometer (Promega). Equal concentrations of amplicons were pooled prior to sequencing.

#### Technologies used

Amplicon libraries' preparation and sequencing were performed at the DNA Sequencing platform of the Institute of Marine Biology, Biotechnology and Aquaculture (IMBBC, HCMR) using *454* GS *FLX* Titanium technology (Roche, *454* Life Sciences), according to the manufacturer's recommendations.

### Data processing

The 454 GS FLX Titanium metabarcoding data were processed in the following steps: a) raw data were denoised using the software package AmpliconNoise v.1.29 (Quince et al. 2011) by removing pyrosequencing noise and correcting for putative PCR errors; b) the Perseus algorithm, included in AmpliconNoise, was used to check for chimeras and the denoised reads were clustered to Operational Taxonomic Units (OTUs), with a complete linkage algorithm on a 97% sequence identity level (Quince et al. 2011); c) taxonomy assignment was performed using GTDB r207 (Parks et al. 2022) using the IDTAXA Classifier (Murali et al. 2018) and a confidence threshold ≥ 60% (cautious). AmpliconNoise and Perseus were run with the default parameters. IDTAXA Classifier was run using the IDTAXA web tool.

The phyloseq package (version 1.42.0) (McMurdie and Holmes 2013) was used to generate bar charts and calculate beta diversity, using Bray-Curtis distances visualised with non-metric multidimensional scaling (nMDS).

#### Technologies used

The analysis was carried out through Zorba, the High Performance Computing (HPC) system of IMBBC (Zafeiropoulos et al. 2021).

## Biodiversity scope

The majority of the reads were unclassified (~ 26%). The most abundant phyla were Proteobacteria (~ 19%), Chloroflexota (~ 9%), Cyanobacteria (~ 8%) and Poribacteria (~ 6%). The processed sequences, along with their corresponding taxonomic information and metadata, can be found in the occurrence dataset available from GBIF. In addition, processed sequences using the MGnify pipeline (4.1) and their respective taxonomic information is available with the MGnify study id MGYS00004687 (available at https://www.ebi.ac.uk/metagenomics/studies/MGYS00004687) (Richardson et al. 2022).

### Target

The target of the dataset was to amplify prokaryotic taxa associated with the studied sponges.

### Taxonomic range

Archaea, Bacteria

## Data Resources

Details for the samples can be found in Suppl. material 1 and Suppl. material 2. All the raw sequence files of this study were submitted to the European Nucleotide Archive (ENA) (Burgin et al. 2022) with the study accession number PRJEB23640 (available at http://www.ebi.ac.uk/ena/data/view/PRJEB23640).

### Resource 1

Download URL: ftp://ftp.sra.ebi.ac.uk/vol1/fastq/ERR220/001/ERR2204961/ERR2204961.fastq.gz

Resource identifier: ERR2204961

Data format : FASTQ

### Resource 2

Download URL: ftp://ftp.sra.ebi.ac.uk/vol1/fastq/ERR248/008/ERR2486828/ERR2486828.fastq.gz

Resource identifier: ERR2486828

Data format : FASTQ

### Resource 3

Download URL: ftp://ftp.sra.ebi.ac.uk/vol1/fastq/ERR248/009/ERR2486829/ERR2486829.fastq.gz

Resource identifier: ERR2486829

Data format : FASTQ

### Resource 4

Download URL: ftp://ftp.sra.ebi.ac.uk/vol1/fastq/ERR248/001/ERR2486831/ERR2486831.fastq.gz

Resource identifier: ERR2486831

Data format : FASTQ

### Resource 5

Download URL: ftp://ftp.sra.ebi.ac.uk/vol1/fastq/ERR248/003/ERR2486833/ERR2486833.fastq.gz

Resource identifier: ERR2486833

Data format : FASTQ

### Resource 6

Download URL: ftp://ftp.sra.ebi.ac.uk/vol1/fastq/ERR248/005/ERR2486835/ERR2486835.fastq.gz

Resource identifier: ERR2486835

Data format : FASTQ

### Resource 7

Download URL: ftp://ftp.sra.ebi.ac.uk/vol1/fastq/ERR248/006/ERR2486826/ERR2486826.fastq.gz

Resource identifier: ERR2486826

Data format : FASTQ

### Resource 8

Download URL: ftp://ftp.sra.ebi.ac.uk/vol1/fastq/ERR248/007/ERR2486827/ERR2486827.fastq.gz

Resource identifier: ERR2486827

Data format : FASTQ

### Resource 9

Download URL: ftp://ftp.sra.ebi.ac.uk/vol1/fastq/ERR248/004/ERR2486834/ERR2486834.fastq.gz

Resource identifier: ERR2486834

Data format : FASTQ

### Resource 10

Download URL: ftp://ftp.sra.ebi.ac.uk/vol1/fastq/ERR248/007/ERR2486837/ERR2486837.fastq.gz

Resource identifier: ERR2486837

Data format : FASTQ

### Resource 11

Download URL: ftp://ftp.sra.ebi.ac.uk/vol1/fastq/ERR248/009/ERR2486839/ERR2486839.fastq.gz

Resource identifier: ERR2486839

Data format : FASTQ

### Resource 12

Download URL: ftp://ftp.sra.ebi.ac.uk/vol1/fastq/ERR248/002/ERR2486842/ERR2486842.fastq.gz

Resource identifier: ERR2486842

Data format : FASTQ

### Resource 13

Download URL: ftp://ftp.sra.ebi.ac.uk/vol1/fastq/ERR248/003/ERR2486843/ERR2486843.fastq.gz

Resource identifier: ERR2486843

Data format : FASTQ

### Resource 14

Download URL: ftp://ftp.sra.ebi.ac.uk/vol1/fastq/ERR248/000/ERR2486830/ERR2486830.fastq.gz

Resource identifier: ERR2486830

Data format : FASTQ

### Resource 15

Download URL: ftp://ftp.sra.ebi.ac.uk/vol1/fastq/ERR248/002/ERR2486832/ERR2486832.fastq.gz

Resource identifier: ERR2486832

Data format : FASTQ

### Resource 16

Download URL: ftp://ftp.sra.ebi.ac.uk/vol1/fastq/ERR248/006/ERR2486836/ERR2486836.fastq.gz

Resource identifier: ERR2486836

Data format : FASTQ

### Resource 17

Download URL: ftp://ftp.sra.ebi.ac.uk/vol1/fastq/ERR248/008/ERR2486838/ERR2486838.fastq.gz

Resource identifier: ERR2486838

Data format : FASTQ

### Resource 18

Download URL: ftp://ftp.sra.ebi.ac.uk/vol1/fastq/ERR248/000/ERR2486840/ERR2486840.fastq.gz

Resource identifier: ERR2486840

Data format : FASTQ

### Resource 19

Download URL: ftp://ftp.sra.ebi.ac.uk/vol1/fastq/ERR248/001/ERR2486841/ERR2486841.fastq.gz

Resource identifier: ERR2486841

Data format : FASTQ

### Resource 20

Download URL: ftp://ftp.sra.ebi.ac.uk/vol1/fastq/ERR248/004/ERR2486844/ERR2486844.fastq.gz

Resource identifier: ERR2486844

Data format : FASTQ

## Results

**Figure 1.**
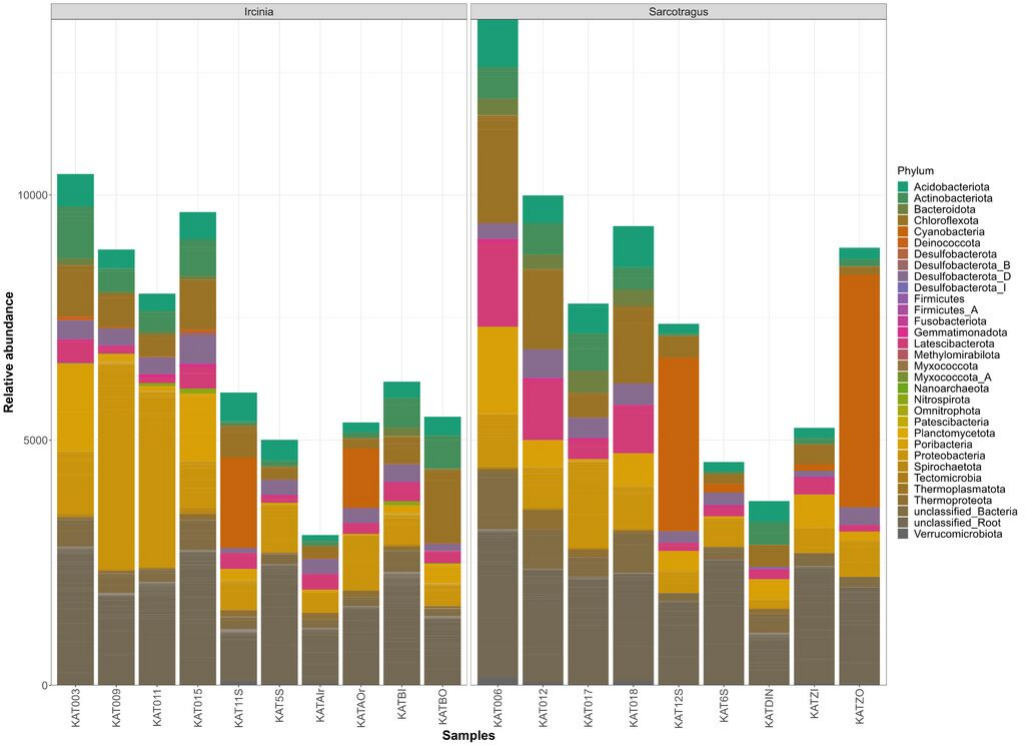
Bar chart showing the abundances of the main microbial taxa, at the phylum level, at each of the sponge samples.

**Figure 2.**
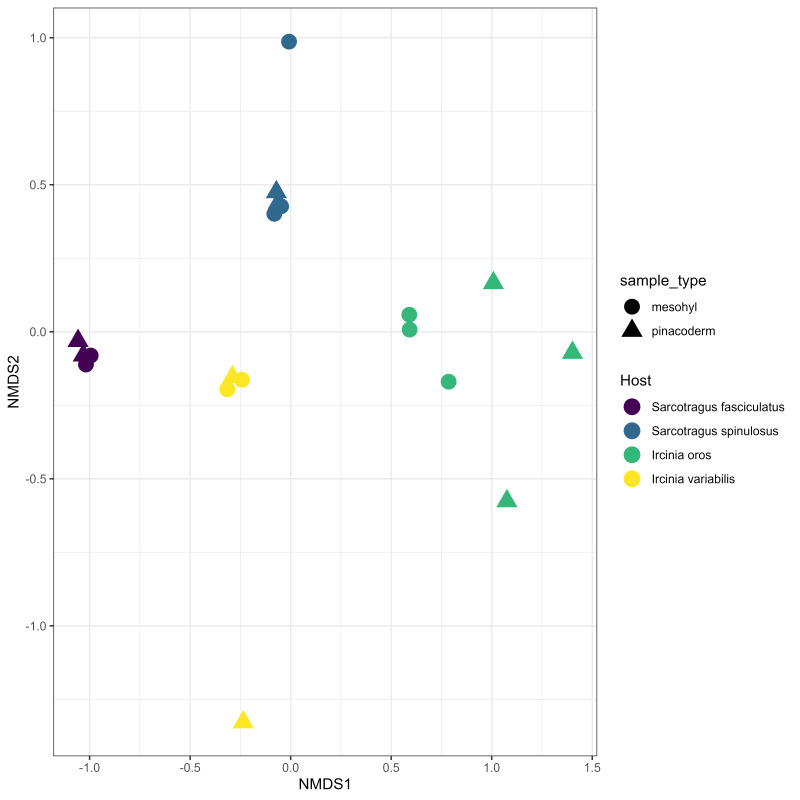
nMDS plot of the Bray-Curtis similarity matrix of the sponge samples, based on the microbial OTUs abundances.

The most abundant phyla across all the samples are Proteobacteria (~ 19%), Chloroflexota (~ 9%), Cyanobacteria (~ 8%) and Poribacteria (~ 6%) (Fig. 1). It seems that the microbial community profiles are clustering, based on the host species (PERMANOVA: R^2^ = 0.56, p = 0.001), but not based on the sampled tissue (PERMANOVA: R^2^ = 0.073, p = 0.23) (Fig. 2).

Results

## Discussion

Since the advent of pyrosequencing, research papers describing and discussing findings, based on eDNA and DNA metabarcoding data, have been a major part of the scientific literature. However, submission of such data into repositories was not possible until very recently, with the development of the Darwin Core (DwC) DNA derived data extension (Finstad et al. 2023). This extension is based on the MIxS checklists (Yilmaz et al. 2011) which have been developed by the Genomic Standards Consortium (GSC) and are the golden standard for describing sample metadata information in a standardised way when submitting raw sequence data to the relevant repositories (e.g. ENA, NCBI etc.). To ensure the interoperability between the different repositories and platforms, as well as between the different metadata standard formats, Biodiversity Information Standards (TDWG) and GSC have agreed on the creation of a continuous model to synchronise their standards; undoubtedly, such efforts on enhancement of data-sharing and utilisation across domains will definitely have a major impact on future scientific discoveries (Meyer et al. 2023).

It should be noted, however, that certain metabarcoding limitations should be taken into account when preparing DwC DNA occurrence data for submission to GBIF and/or OBIS. Notably, the uncertainty of organism occurrences, along with the less precise taxonomic identifications inferred from sequencing data, should be clearly mentioned in the dataset descriptions. Moreover, MIxS checklists in host-associated datasets, while capturing the host species information in the sample metadata, fail to do so in the run, i.e. sequence, metadata; an example would be this particular dataset, where the taxonomy associated with the run accession numbers in ENA is "sponge metagenome", with no further details shown, although the host species is mentioned in the sample metadata, i.e. it can be retrieved when searching ENA with the sample accession number. In addition, standards should be continuously updated to incorporate changes in sequencing technologies and protocols, in order to aid researchers document such data in a more FAIR way. If discrepancies between repositories and platforms are minimised and even eliminated in the future, the already high intrinsic value of DNA derived datasets will increase even further.

Discussion

## Usage Rights

CC BY 4.0

Usage Rights

## Supplementary Material

4300FFA2-5DC4-5967-BD40-6EDBE93B66D7 10.3897/BDJ.12.e114809.suppl1 Supplementary material 1 BioSamples MIxS checklists Data type sample metadata File: oo_909999.csv https://binary.pensoft.net/file/909999 HELLENIC CENTRE FOR MARINE RESEARCH

CC4FC557-B4A0-50AC-A304-7FD45ECC500B 10.3897/BDJ.12.e114809.suppl2 Supplementary material 2 Sample metadata Data type metadata File: oo_957028.txt https://binary.pensoft.net/file/957028 Katerina Skaraki, Christina Pavloudi

## Figures and Tables

**Table 1.** Locality, geographical coordinates and depth (m) of the sampling stations.

**Locality**	**Coordinates**	**Depth (m)**
Alikes	35.41568N, 24.9881E	6-10
Elounda	35.2521N, 25.7592E	12-14
